# The Interplay Between Carotid Intima-Media Thickness and Selected Serum Biomarkers in Various Stages of Chronic Kidney Disease

**DOI:** 10.3390/biomedicines13020335

**Published:** 2025-02-01

**Authors:** Mateusz Twardawa, Piotr Formanowicz, Dorota Formanowicz

**Affiliations:** 1 Institute of Computing Science, Poznan University of Technology, 60-965 Poznan, Poland; mateusz.twardawa@cs.put.poznan.pl (M.T.); piotr.formanowicz@cs.put.poznan.pl (P.F.); 2 ICT Security Department, Poznan Supercomputing and Networking Center Affiliated to the Institute of Bioorganic Chemistry, Polish Academy of Sciences, 61-139 Poznan, Poland; 3 Department of Medical Chemistry and Laboratory Medicine, Poznan University of Medical Sciences, 60-806 Poznan, Poland

**Keywords:** atherosclerosis, IMT, oxidative stress, chronic kidney disease, general linear models

## Abstract

**Background/Objectives**: Chronic kidney disease (CKD), the most common cause of which is hypertension and diabetes, is a recognized risk factor for cardiovascular disease (CVD). This study investigated the association between selected serum biomarkers in the context of intima-media thickness (IMT) changes, a common predictor of subsequent cardiovascular (CV) events. **Methods**: A total of 251 individuals were enrolled in the study, divided into groups based on the severity of CKD, the presence of CVD, and healthy controls. For this purpose, the data from the following groups of participants were analyzed: (1) end-stage renal disease (ESRD) (n = 106), (2) pre-dialyzed (PRE) (n = 48), (3) patients at stages 1 and 2 of CKD (CKD1-2) (n = 37), (4) patients with CVD and no kidney disease (CARD) (n = 28), and (5) healthy controls (HV) (n = 31). To find markers associated with elevated IMT, the each group with CVD (ESRD, PRE and CARD) was separated into two subgroups with normal and elevated IMT and compared in the relation of the studied serum biomarkers. **Results**: The findings identified glucose as the only marker exclusively associated with CVD. Markers uniquely linked to CKD included urea, creatinine, eGFR, total protein, CEL, neopterin, total calcium, phosphates, iPTH, sodium, iron, ferritin, and AST. All other markers reflected a combined influence of both CKD and CVD. By comparing patients with normal and elevated IMT, distinct types of CKD–CVD interactions were observed, i.e., independent (additive effects of CKD and CVD) for MPO, ALP, MMP-9, and MMP-9/TIMP-1; combined (enhanced effect due to interactions) for AOPPs and TIMP-1; and conditional (CVD impact specific to CKD patients) for AGEs, 3-NT, magnesium, UIBC, TIBC, ALT, and TIMP-1/MMP-9. However, certain markers, i.e., CML, sRAGEs, carbamylated protein groups, protein carbamylation, hsCRP, TC, HDL-C, LDL-C, TG, IL-18, klotho, FGF-23, klotho/FGF-23 ratio, potassium, NT-proBNP, and AIP were associated with both CKD and CVD, though the exact nature of their interaction could not be determined using IMT as a distinguishing factor. **Conclusions**: The results showed that relations between IMT and the remaining studied factors were not trivial, and most of the analyzed parameters were altered in CKD patients, especially if compared to patients with CVD but without CKD. IMT cannot be used as a universal CVD marker.

## 1. Introduction

Atherosclerosis is a serious global health problem, especially harmful for patients with impaired kidney function. Since chronic kidney disease (CKD) is one of the fastest-increasing causes of death in the world and is expected to be in the top five causes by 2040 [1], all factors that could contribute to a worsening prognosis are particularly important to address.

Since the leading causes of CKD are diabetes and hypertension, which are also risk factors for atherosclerosis, there is a direct relationship between atherosclerosis and a reduced glomerular filtration rate (GFR) and albuminuria, which are the basis for the diagnosis of CKD.

Moreover, increased oxidative stress characterizes CKD and progressive renal dysfunction. As is known, oxidative stress is a cumulative factor associated with the loss of renal function and/or the type of renal replacement therapy in dialyzed patients. In patients with CKD, oxidative stress is, in some sense, a consequence of increased reactive oxygen species (ROS) generation. At the same time, impaired clearance of pro-oxidants due to renal function impairment and an ineffective antioxidant defense system play an additional role. Importantly, oxidative stress and chronic inflammation are critical components of CKD-related pathologies, which can adversely affect the human body, leading to many disorders [2].

Taking into account numerous studies also conducted by the authors, e.g., [3,4,5,6,7], various types of atherosclerosis can be distinguished: (1) the so-called classic type, associated with well-known cardiovascular (CV) risk factors but without concomitant CKD, or with concomitant CKD, but with a predominant impact of traditional risk factors, and (2) the type referred to as non-classic type, with CKD as the determining factor. These types of atherosclerosis are characterized by different grades of atherosclerotic plaque development and the degree of its advancement, revealing that other processes underline them. It is enough to look at the structure of atherosclerotic plaque in a patient with CKD to notice that they contain more calcium deposits in their inner part. This calcification makes the plaque harder and more fragile, increasing the risk of CV events. The content of collagen fibers and smooth muscle cells is also reduced compared to plaques in patients with classic CV risk factors.

To summarize, atherosclerotic plaque in a patient with CKD carries baggage resulting from CKD, the impact of which changes and intensifies along the deterioration of kidney function. Moreover, it is influenced by factors accompanying CVD, typically found in patients suffering from CVD without clinical or laboratory signs of CKD. Interestingly, the effect of well-known factors such as dyslipidemia is weak in CKD, and an inverse association is even mentioned here. However, in CKD, there are other factors such as calcium and phosphate metabolism disorders, hypervolemia, anemia, intravenous iron therapy, the influence of uremic toxins, and dialysis procedures; all these promote and deepen the oxidative stress and the inflammatory process, increasing their participation in the processes taking place in the subendothelial layer of the vessels [8]. Due to the multitude of factors contributing to atherosclerosis among CKD patients, they are at increased risk of developing CVD [2,9].

Atherosclerotic plaque build-up is a dynamic process in which the existing thickness depends on the thickness of the previous stage [10]. In the clinical setting, carotid intima-media thickness (IMT) (a structure of the arterial wall that extends from the border of the adventitia and tunica media to the border of the tunica media and the vessel’s lumen) is regarded as a proxy for plaque development and patient survival.

Hence, this study explored the relationships between selected basic metabolic, oxidative stress, inflammatory, lipid, metalloproteinase, calcium–phosphate balance, ion, iron, and cardiovascular (CV) risk-related serum biomarkers in detail, in the context of IMT changes among various groups of patients differing in kidney function and treatment. Assuming that IMT serves as a reliable prognostic marker for CV risk, patients were divided into subgroups with normal and elevated IMT and compared to identify the type of CVD–CKD relationship (independent, combined, or conditional) associated with the analyzed serum markers.

## 2. Materials and Methods

### 2.1. Study Group

This is another article by the authors based on the conducted cross-sectional case-control study, where 251 participants with diagnosed CKD (n = 183) and without CKD (n = 68) met the required criteria and participated (ClinicalTrials.gov Identifier: NCT05214872). The recruitment for the study lasted two years.

During the 2-year follow-up period from the study entry, the medical history of fatal CV events was recorded individually for each participant. The primary endpoint was fatal acute myocardial infarction (AMI), acute ischemic stroke, or any sudden death if the autopsy results confirmed a CV association. If there was a death of a participant within two years of study entry, but the cause of death could not be defined, these patients were excluded and not further considered.

Based on the presence or absence of CKD, the stage of CKD, and the type of renal replacement therapy used, five study groups were created, where eGFR was calculated following the recommendations (KDIGO 2012) according to the MDRD formula based on serum creatinine concentration [mg/dL]. Since multiple criteria were considered while segregating patients into groups, the final decision on group assignment was in all cases based on the patient’s official diagnosis made by a medical professional.

**Figure 1:**
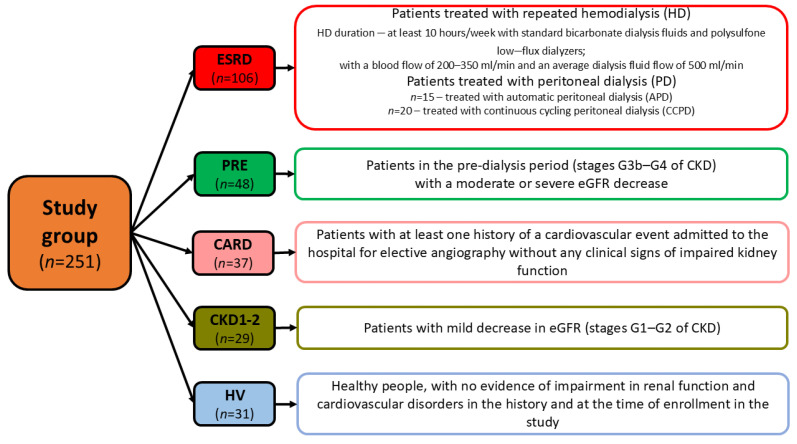
General characteristics of the groups of patients recruited in this study.

These groups were as follows: (1) patients with end-stage renal disease (ESRD) with a minimum of 6 months of repeated dialysis treatment; (2) pre-dialyzed (PRE) patients, (3) patients with CVD but without CKD (CARD); (4) patients with stage 1 and 2 CKD (CKD1-2), and (6) healthy volunteers (HV); for details see Figure 1.

The CKD patients were recruited from the Poznan Clinical Hospital H. Święcicki (Nephrology Outpatient Clinic, Peritoneal Dialysis Clinic, and the Dialysis Center).

Patients assigned to the CARD group had a history of at least one CV event. They were admitted to the Department of Intensive Care of Cardiology and Internal Diseases at Poznan Clinical Hospital H. Święcicki for elective angiography with no signs of renal dysfunction.

The control group (HV) consisted of healthy people with no evidence of renal function impairment and CVD in their history and at the study’s enrollment time. This group was recruited from among people reporting to the laboratory for routine check-ups.

This group selection was intended to track changes in IMT and differences between individual stages of CKD.

Our assumption was to treat CKD as a CVD-related disease. Thus, we included in this study the CKD1-2 group, where CKD accompanied by CVD was just beginning, then PRE group, including more advanced kidney disease + more advanced CVD, and finally, the group of dialyzed patients (ESRD), containing patients with the most advanced stage of CKD, that were treated with hemodialysis or peritoneal dialysis.

At the same time, we wanted to obtain results from a group that suffered from CVD but did not have laboratory or clinical signs of CKD (CARD group) to compare the impact of CVD on IMT if there was no kidney disease. The second reference group was a group of healthy volunteers (HV) matched according to age, to observe the impact of only age on the studied serum biomarkers.

Exclusion criteria for all study participants were: (1) active acute infection, (2) history of immunosuppressive treatment, (3) kidney transplant, (4) abnormal liver function, (5) malignancy within the last five years, and (6) alcohol abuse within the last five years.

### 2.2. Basic Metric Information of the Studied Participants

Basic metric information showing differences between the groups is provided for the following parameters: age, percent of females, percent of smoking patients, body mass index (BMI) [kg/m^2^] which was calculated by dividing a person’s body weight (post-dialysis body weight in the ESRD group) [kg] by the square of participants’ body height [m], percent of overweight patients, percent of deceased patients in the 2-year follow-up, duration of dialysis treatment, and a mean number of months lived for patients who died during the 2-year follow up.

### 2.3. Laboratory Parameters and Procedures

At the time of enrollment in the study, all subjects had blood samples taken for laboratory tests, and the sample collection procedure was identical for all study participants. Blood collection from dialysis patients had to be synchronized with the dialysis schedule and the week when routine laboratory tests were planned. In this group, if patients were treated by hemodialysis (HD), blood samples were collected in the morning (before heparin administration) before the second HD procedure in the week. All samples were stored at −80 °C and processed similarly.

Analyses of individual parameters were constantly carried out by the same team and in similar atmospheric conditions. All studied laboratory parameters were performed using standard methods following the manufacturer’s recommendations. For the current study, in accordance with the assumed goal, the following parameters were used:

- Glucose (Glu) [mg/dL], urea [mg/dL], creatinine [mg/dL], uric acid [mg/dL], and total protein (TP) [g/dL]; lipid profile: (a) total cholesterol (T-C) [mg/dL]; (b) high-density lipoprotein cholesterol (HDL-C) [mg/dL]; and (c) triglycerides (TG) [mg/dL] were assessed by routine techniques using an automated Cobas Integra 400 plus biochemical analyzer, Roche Diagnostics, Indianapolis, Indiana, USA; and (d) low-density lipoprotein cholesterol (LDL-C) [mg/dL] was determined from Friedewals’ formula (LDL-C [mg/dL] = total cholesterol (T-C) [mg/dL] − HDL-C [mg/dL] − TG [mg/dL]/5); (e) estimated glomerular filtration rate (eGFR) was calculated following the recommendations (KDIGO 2012) according to the MDRD formula based on the serum creatinine concentration [mg/dL]: estimated eGFR = 186 × (creatinine serum concentration [mg/dL]) − 1.154 × (age) [years] − 0.203 (for women, the result was multiplied by 0.724).
- Serum markers of inflammation: (a) neopterin [nmol/L] was assessed by the ELISA method, using DRG International Inc. kits, Springfield, New Jersey, USA; (b) interleukin 18 (IL-18) [pg/mL] was determined by using Colorimetric Sandwich ELISA, Quantikine Human IL-18 R&D Inc., Toronto, ON, Canada; (c) high-sensitivity C-reactive protein (hsCRP) [mg/L] was measured using a high-sensitivity analyzer (Behring Nephelometer Analyzer II, Dade Behring Inc., Deerfield, IL, USA).
- Serum markers of oxidative stress: (a) advanced oxidation protein products (AOPPs) [µmol/mg protein] were assessed by the ELISA method for AOPPs, using Shanghai Sunred Biological Technology Co. kit, Shanghai, China; (b) advanced glycation end products (AGEs) [µg/mg protein] were assessed by the ELISA method for AGEs, using Shanghai Sunred Biological Technology Co. kit, Shanghai, China; (c) carboxymethyl^*ϵ*^(lysine) (CML) [µg/mg protein] was assessed by the ELISA method for CML, using Shanghai Sunred Biological Technology Co. kits, Shanghai, China; (d) 3-nitrotyrosine (3-NT) [µmol/mg protein] was assessed by the ELISA method for 3-NT, using Shanghai Sunred Biological Technology Co. kit, Shanghai, China; (e) methylglyoxal (MG) [µg/mg protein] was assessed with the competitive ELISA method, using MG kit from Cell Biolabs Inc., San Diego, CA, USA; (f) carboxyethyl^*ϵ*^(lysine) (CEL) [µg/mg protein] was assessed by the competitive ELISA method using CEL kits from Cell Biolabs Inc., San Diego, CA, USA; (g) carbamyl groups of proteins (CBL-BSA) [µg/mg protein] were assessed by the competitive ELISA method using the carbamyl protein groups kit from Cell Biolabs Inc., San Diego, CA, USA; (h) soluble receptor for advanced glycation end products (sRAGEs) [µg/mg protein] were assessed by the ELISA method using R&D Systems sRAGE kit, Toronto, ON, Canada; (i) myeloperoxidase (MPO) [ng/mL] was assessed by the ELISA method using the Quantikine Human MPO test by R&D Systems kit, Toronto, ON, Canada.
- Serum markers of calcium and phosphate metabolism: total calcium, phosphate (${\mathrm{PO}}_{4}^{3-}$), and intact parathyroid hormone (iPTH) were assessed by routine techniques using the Cobas Integra 400 plus biochemical analyzer from Roche Diagnostics, Indianapolis, Indiana, USA.
- Serum selected electrolytes: magnesium (Mg^2+^) [mg/dL], sodium (Na^+^) [mmol/L], and potassium (K^+^) [mmol/L] concentrations were assessed by the routine techniques using the Cobas Integra 400 plus biochemical analyzer by Roche Diagnostics, Indianapolis, IN, USA.
- Serum liver enzymes activity: alkaline phosphatase (ALP) [U/L], alanine transaminase (ALT) [U/L], and aspartate transaminase (AST) [U/L], were assessed by the routine techniques using the Cobas Integra 400 plus biochemical analyzer by Roche Diagnostics, Indianapolis, IN, USA.
- Serum iron metabolism selected markers: iron (Fe) concentration [mg/dL], total iron-binding capacity (TIBC), ferritin [ng/mL] in the serum were assessed with the Cobas Integra 400 plus biochemical analyzer from Roche Diagnostics, Indianapolis, Indiana, USA; unsaturated iron-binding capacity (UIBC) [mg/dL] was determined by an equation in which iron [mg/dL] concentration in plasma was subtracted from TIBC [mg/dL].
- Klotho (KL) [ng/mL] serum concentration was analyzed by the Human KL (Klotho) [ng/mL] ELISA Kit, Shanghai Sunred Biological Technology Co. kit, Shanghai, China.
- Fibroblast growth factor 23 (FGF-23) [pg/mL] serum concentration was analyzed using the Human FGF-23 ELISA Kit, Sigma-Aldrich, MA, USA.
- N-terminal pro-B-type natriuretic peptide (NT-proBNP) [fmol/mL] was analyzed by enzyme immunoassay (EIA) using the Nt-proBNP kit from Biomedica, Dúbravka, Slovakia.
- The Atherogenic Index of Plasma (AIP) was calculated as $log_{10}\left(\frac{\mathrm{TG}\;\left[\mathrm{mmol}/L\right]}{\mathrm{HDL}-C\;\left[\mathrm{mmol}/L\right]}\right)$ [11].

### 2.4. IMT Assessment

Using a standardized methodology, the same person measured the carotid IMT by an Accuson CV 70 system (Siemens, Germany) with a 10 MHz transducer. Two longitudinal views (anterolateral and posterolateral) were assessed. The distal 1 cm of the common carotid artery proximal to the bulb was measured using a computer analysis system (Medical Imaging Applications, LLC., Coralville, IA, USA).

### 2.5. Separation of Patients into Subgroups Based on IMT

The Shapiro–Wilk test was performed along with a visual inspection to check whether the IMT distribution was normal within each group. If a non-normal distribution was revealed and there was a sufficient number of records in the data, it was possible to separate patients with normal (expected) IMT levels from patients with elevated IMT levels. Patients in the ESRD, PRE, and CARD groups were classified into two subgroups with postfix n in their names (patients with normal IMT levels) and postfix e (patients with elevated IMT levels) based on IMT level with respect to age. The values used for the selection process were taken from a large population study by Engelen et al. [12]. Since dialyzed and cardiovascular patients are expected to have a significantly higher IMT than the general population, the selection boundaries were established on the 97.5th percentile for each age interval. The exact values of age-related thresholds are summarized in Table 1.

### 2.6. Statistical Analysis and Modeling

The majority of statistical analyses and visualizations were performed in Python and involved popular modules, i.e., numpy [13], scipy [14], matplotlib [15], pandas [16], and seaborn [17]. The exception was the analysis involving general linear models, which was executed in R with the use of lme4 [18] and sjPlot [19] libraries. $\chi^{2}$ and Kruskal–Wallis H tests were used to study general differences between the groups.

The initial analysis of IMT was based on Shapiro–Wilk’s test and visual inspection. Two additional subgroups (with normal and elevated IMT) were created for each of the following groups: ESRD, PRE, and CARD. In the CKD1-2 and HV groups, there were no patients with elevated IMT; hence, these groups were not divided.

Patients were segregated based on age-specific thresholds for IMT levels that were set to the 97.5th percentile of the general population (see [12] for more information on referential IMT levels).

The analyses of all included serum markers and calculated indices were performed to detect potential factors that could be related to elevated IMT (postfix “e”) in the studied groups in comparison to not-elevated IMT (postfix “n”).

All the subgroups pairs (ESRDn vs. ESRDe, PREn vs. PREe, CARDn vs. CARDe) were compared using Mann–Whitney U tests.

To model the hypothetical influence of CVD and CKD on the analyzed serum biomarkers, three additional general linear models (GLMs) were developed for each studied statistical hypothesis involving the interplay of CVD and CKD: 1—sole effect of CKD, 2—sole effect of elevated IMT, 3—independent relationship with kidney disease and elevated IMT (simple additive effect), 4—mutual combination of CKD and elevated IMT effects, 5—mixed effect model showing the impact of CKD on elevated IMT levels. The GLM analysis provided additional guidance for the results obtained, supporting the existing statistical analyses. All of the results produced by GLM analyses are placed in Supplementary Materials (Tables S1–S5).

The relationships between CVD and CKD in the context of the analyzed markers were determined in the following fashion. At first, the relationship with CKD in the form of differences between HV, CKD1-2, PRE, and ESRD groups was determined. If CKD groups differed significantly from HV and/or between each other group, the relation with CKD was confirmed. A comparison of the CARD group with HV and patients with different stages of CKD provided information on marker relevance in the context of cardiovascular issues. Nonetheless, that information was not enough to determine the nature of the relationship between CKD and CVD. For this reason, if noticeable dissimilarities were detected between pairs of subgroups defined by the IMT level, a further analysis was performed. If the whole CARD group was not showing differences with HV, detecting differences between groups with normal and elevated IMT was enough to assign a relation to CVD to a marker. The following types of interplay between elevated IMT and CKD were analyzed in this work: the independent contribution of CKD and elevated IMT, the combined effect of IMT and CKD, and the conditional effect of IMT depending on CKD stage or, in other words, CVD markers that were unique to renal dysfunction. This step was based on previously executed statistical tests and GLMs designed for this purpose.

**Figure 2:**
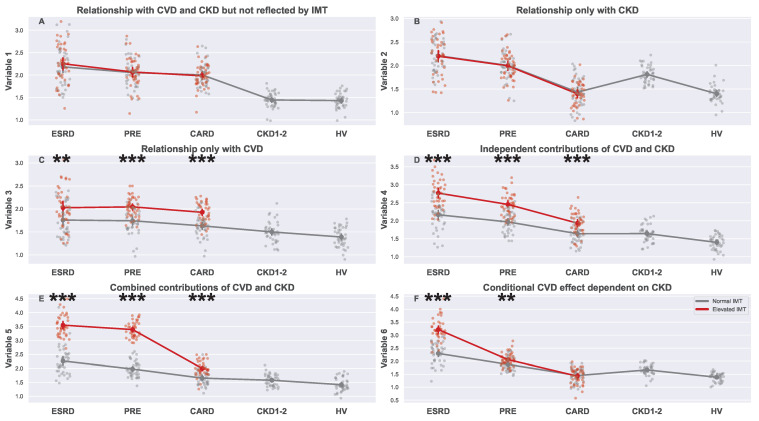
Strip plots showing hypothetical dependencies between serum markers (or other parameters) and the studied diseases: CKD and CVD. The plots were generated using data sampled from a normal distribution to illustrate how various types of relationships between variables and diseases may appear. The figure presents six scenarios: (**A**) The variable is not related to either CKD and CVD, unlike IMT; therefore, the nature of the interplay between CKD and CVD cannot be deduced; (**B**) the variable level is associated only with CKD; (**C**) the variable level is associated only with CVD; (**D**) both CVD and CKD independently affect the variable; (**E**) both CVD and CKD influence the variable, with a stronger effect when they co-occur; (**F**) the influence of CVD on the variable is present only when the patient also has CKD (conditional dependence). Each plot displays five main groups: ESRD, PRE, CARD, CKD1-2, and HV. Subgroups with normal and elevated IMT are distinguished using different colors (gray for normal and red for elevated). For each subgroup pair (ESRDn vs. ESRDe, PREn vs. PREe, and CARDn vs. CARDe) and the undivided groups (CKD1-2 and HV), the mean is represented by a more saturated dot, with error bars indicating the standard deviation for each group or subgroup. Note that mean values for the entire ESRD, PRE, and CARD groups are not provided. Additional lines connecting the means are included to enhance plot readability. The pairs of subgroups with normal and elevated IMT readings were annotated with the Mann–Whitney U test significance level (**: 0.01 > *p*-value ≥ 0.001, ***: *p*-value < 0.001).

Figure 2 was added as a help for the results’ interpretation. In this study, various relationships between a serum marker and CVD or CKD could be detected; the most typical included no relationship with the IMT, but existing relations with CVD and CKD (Figure 2A), parameter related only to kidney disease (Figure 2B), parameter related only to CVD (Figure 2C), the parameter is independently related to kidney disease and CVD creating simple additive effect (Figure 2D), a combined effect of kidney disease and CVD (Figure 2E), and unique changes in serum marker levels related to CVD, which develop only in CKD patients (Figure 2F).

## 3. Results

### 3.1. General Information

**Figure 3:**
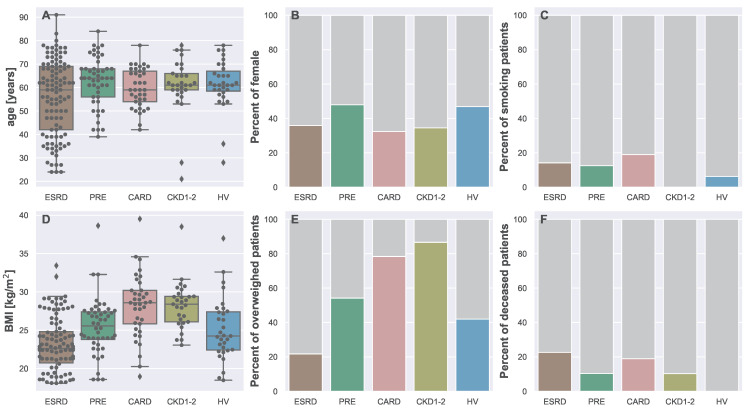
Differences between the groups (ESRD, PRE, CARD, CKD1-2, and HV) analyzed in this study. The figure is made out of six subplots: (**A**)—age variety within the groups, (**B**)—differences in the percent of females within the groups, (**C**)—differences in the percent of smokers, (**D**)—BMI of patients from different groups, (**E**)—differences in the percent of overweight patients within the groups, (**F**)—differences in the percent of deceased patients during the two-year follow up.

Basic information about the groups is presented in Figure 3 and Table 2. As shown in plot A in Figure 3, age did not differ significantly between the groups (Kruskal–Wallis H = 6.8282, *p* = 0.1452). Visualized in Figure 3B, the percent of females was similar in each group ($\chi^{2}$ = 5.4114, *p* = 0.2472); however, smokers were unevenly distributed between the groups ($\chi^{2}$ = 20.8837, *p* < $10^{-4}$), which can be observed in Figure 3C. Body mass index (Kruskal–Wallis H = 65.67, *p* < $10^{-4}$) as well as percent of overweight patients ($\chi^{2}$ = 49.7612, *p* < $10^{-4}$) differed between the groups as can be seen in Figure 3D,E, respectively. The highest percent of deceased patients, as visualized in Figure 3F and given in Table 2, was in the ESRD group ($\chi^{2}$ = 24.8132, *p* < $10^{-4}$). The mean duration of dialysis treatment shown in Table 2 was 19.63 ± 22.64 months.

### 3.2. Separation of Patients Based on IMT

**Figure 4:**
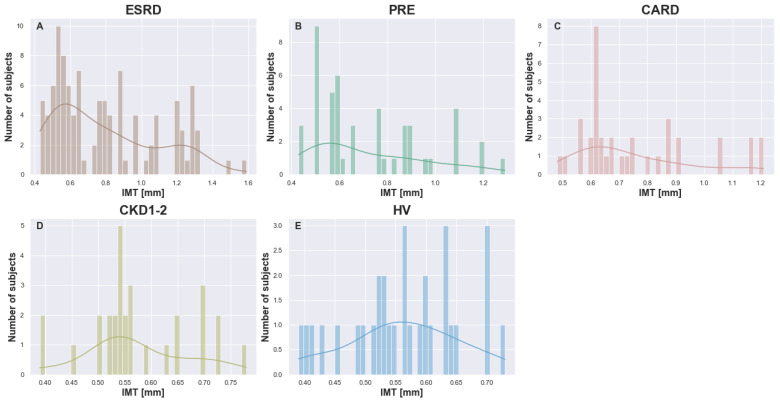
Histograms with overlaid data-derived distributions for IMT [mm] for each analyzed group ((**A**) ESRD, (**B**) PRE, (**C**) CARD, (**D**) CKD1-2, and (**E**) HV).

The IMT differed significantly between the groups (Kruskal–Wallis H = 32.7137, *p*-value $<10^{-4}$). As shown in Figure 4, which presents IMT distributions for the studied groups, the IMT histograms varied between the groups and revealed distinct patterns. The control group had an IMT distribution that was the most similar to the normal one (Shapiro–Wilk W = 0.9732, *p*-value = 0.6119). The same could not be said about other distributions. Groups ESRD (Shapiro–Wilk W = 0.9013, *p*-value = $<10^{-4}$), PRE (Shapiro–Wilk W = 0.9011, *p*-value = 0.0007), CARD (Shapiro–Wilk W = 0.8705, *p*-value = 0.0005), and CKD1-2 (Shapiro–Wilk W = 0.935, *p*-value = 0.0742) showed long tail distributions. However, in the case of ESRD, the plot of the IMT distribution (Figure 4A) had two visible peaks. This may suggest that the ESRD group (and possibly other groups) was heterogeneous and could be separated into two subgroups.

**Figure 5:**
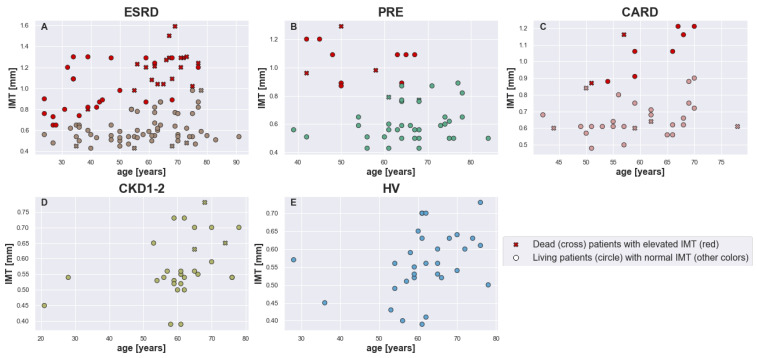
Visualization of separation process for each group ((**A**) ESRD, (**B**) PRE, (**C**) CARD, (**D**) CKD1-2, and (**E**) HV). Patients with elevated IMT are marked with red color. The marker shape denotes whether a patient died (cross) or survived (circle) during the two-year follow-up.

Since the thickness of artery walls grew over the years, the separation of patients with normal and elevated IMT was accomplished according to age-dependent reference tables [12]. The detailed division criteria are placed in Table 1. The division of patients is visualized in Figure 5. Table 3 summarizes the obtained subgroups’ sizes. There was no record of a patient with elevated IMT within the CKD1-2 and HV groups.

### 3.3. Differences Between the Groups and Subgroups with Normal and Elevated IMT

All the groups analyzed in this study were compared based on multiple serum marker levels. This comparison aimed to identify which markers may be related to elevated IMT levels within the groups, and whether such relationships were specific to a given group or universal among all of them. Since the study participants represented various stages of CKD and CVD, their serum biomarkers differed significantly, as shown in Table 4, which presents the results of the Kruskal–Wallis test for all the parameters analyzed in this study. It can be easily spotted that almost all the results showed that parameters were not constant among the groups. Table 4 also contains information on the markers’ relationships with CKD, CVD, and IMT. If all three relations could be assigned to a marker, a type of interplay could be determined (independent, combined, or conditional). If only one type of relationship was determined (with CKD or with CVD in addition with IMT), the relationship was labeled as singular.

**Figure 6:**
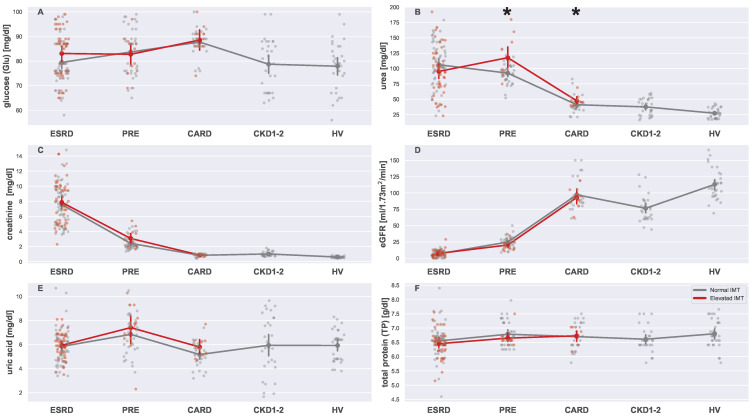
Strip plots presenting differences in levels of serum basic metabolic markers ((**A**) glucose, (**B**) urea, (**C**) creatinine, (**D**) eGFR, (**E**) uric acid, and (**F**) total protein). Each plot displays five main groups: ESRD, PRE, CARD, CKD1-2, and HV. Subgroups with normal and elevated IMTs are distinguished using different colors (gray for normal and red for elevated). For each subgroup pair (ESRDn vs. ESRDe, PREn vs. PREe, and CARDn vs. CARDe) and the undivided groups (CKD1-2 and HV), the mean is represented by a more saturated dot, with error bars indicating the standard deviation for each group or subgroup. Note that mean values for the entire ESRD, PRE, and CARD groups are not provided. Additional lines connecting the means are included to enhance plot readability. The pairs of subgroups with normal and elevated IMT readings were annotated with the Mann–Whitney U test significance level (*: 0.05 > *p*-value ≥ 0.01).

Six basic serum metabolic markers were analyzed in this study., i.e., glucose, urea, creatinine, eGFR, uric acid, and total protein. The results of the comparison of these markers are depicted in Figure 6, which contains six strip plots (one for every serum metabolic marker) and the significance level of the compared types of groups divided based on the IMT and age (with normal and elevated IMT). The plot in Figure 6A presents glucose levels among the analyzed groups. As was presented in Table 4, glucose levels varied significantly between the groups; however, no significant difference was detected while comparing patients with normal and elevated IMTs within each group. Interestingly, dialyzed patients (ESRDn vs. ESRDe) differ visually in the plot, suggesting that an elevated IMT may be connected to higher blood sugar levels. It is well known that diabetes contributes to CVD [20]. Indeed, the CARD patients exhibited the highest blood sugar levels. Nonetheless, the difference was not reflected in the IMT. Moreover, the Mann–Whitney U test did not support the hypothesis that ESRDn and ESRDe differed in glucose levels (Mann–Whitney U = 704, *p*-value = 0.3012).

The differences in urea levels are visualized in Figure 6B. For this marker, significant differences existed between groups PREn vs. PREe (Mann–Whitney U = 288.5, *p*-value = 0.0237) and CARDn vs. CARDe (Mann–Whitney U = 189, *p*-value = 0.0267). Urea and uric acid levels seemed to be slightly related to CVD despite a clearly visible relationship with CKD (lower values for ESRD patients may be connected to treatment effectiveness). The remaining plots (Figure 6C–F) for creatinine, eGFR, uric acid, and total protein, respectively, did not reveal any significant difference between the groups related to normal and elevated IMTs. Creatinine and eGFR presented almost textbook images of CKD dependence (see Figure 2B for a reference). Complete results of all Mann–Whitney U tests are summarized in Table A1.

**Figure 7:**
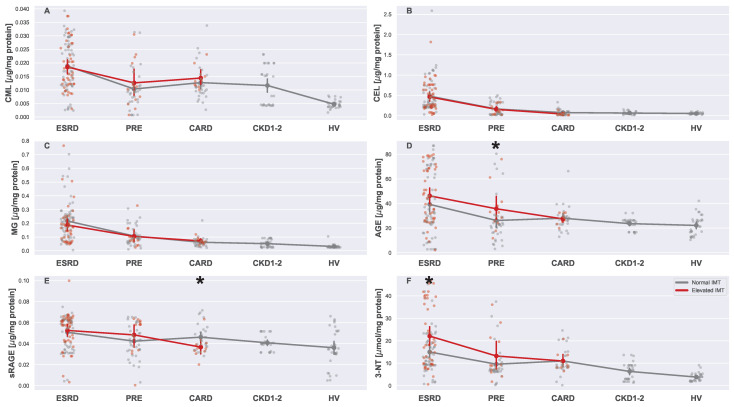
Strip plots presenting differences in levels of serum markers of oxidative stress ((**A**) CML, (**B**) CEL, (**C**) MG, (**D**) AGEs, (**E**) sRAGEs, and (**F**) 3-NT). Each plot displays five main groups: ESRD, PRE, CARD, CKD1-2, and HV. Subgroups with normal and elevated IMTs are distinguished using different colors (gray for normal and red for elevated). For each subgroup pair (ESRDn vs. ESRDe, PREn vs. PREe, and CARDn vs. CARDe) and the undivided groups (CKD1-2 and HV), the mean is represented by a more saturated dot, with error bars indicating the standard deviation for each group or subgroup. Note that mean values for the entire ESRD, PRE, and CARD groups are not provided. Additional lines connecting the means are included to enhance plot readability. The pairs of subgroups with normal and elevated IMT readings were annotated with the Mann–Whitney U test significance level (*: 0.05 > *p*-value ≥ 0.01).

**Figure 8:**
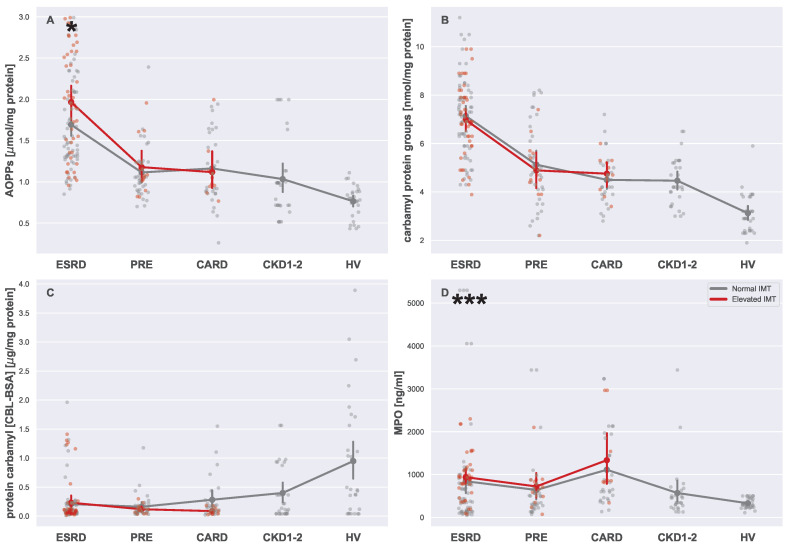
Strip plots presenting differences in serum markers of oxidative stress ((**A**) AOPPs, (**B**) carbamyl protein groups, (**C**) protein carbamyl, and (**D**) MPO). Each plot displays five main groups: ESRD, PRE, CARD, CKD1-2, and HV. Subgroups with normal and elevated IMTs are distinguished using different colors (gray for normal and red for elevated). For each subgroup pair (ESRDn vs. ESRDe, PREn vs. PREe, and CARDn vs. CARDe) and the undivided groups (CKD1-2 and HV), the mean is represented by a more saturated dot, with error bars indicating the standard deviation for each group or subgroup. Note that mean values for the entire ESRD, PRE, and CARD groups are not provided. Additional lines connecting the means are included to enhance plot readability. The pairs of subgroups with normal and elevated IMT readings were annotated with the Mann–Whitney U test significance level (*: 0.05 > *p*-value ≥ 0.01, ***: *p*-value < 0.001).

Variability in serum oxidative stress markers levels is visualized in Figure 7 and Figure 8. Similarly to previous figure, Figure 6, Figure 7 and Figure 8 consist of strip plots dedicated to each oxygen marker that was studied in this work. As can be seen in the figures, CML (Figure 7A), CEL (Figure 7B), MG (Figure 7C), carbamyl protein groups (Figure 8B), and protein carbamyl (Figure 8C) did not differ in patients with normal and elevated IMTs. However, as it can be clearly seen, these parameters were indeed distinct in different groups (see Table 4). Some oxidative stress markers showed a relation dependent only on CKD (CEL, MG, carbamyl protein groups), while MPO seemed to be related to CVD and CKD independently. Interestingly enough, 3-NT, AOPPs, and AGEs seemed to be elevated only for CKD patients with a high IMT, revealing that more complex interplay (conditional: 3-NT, AGEs or combined: AOPPs) was plausible in this case.

**Figure 9:**
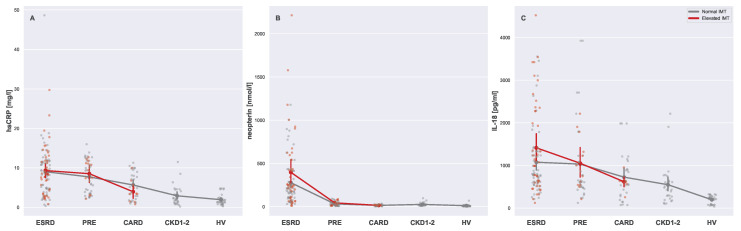
Strip plots presenting differences in serum markers of inflammation (**A**) hsCRP, (**B**) neopterin, and (**C**) IL-18). Each plot displays five main groups: ESRD, PRE, CARD, CKD1-2, and HV. Subgroups with normal and elevated IMTs are distinguished using different colors (gray for normal and red for elevated). For each subgroup pair (ESRDn vs. ESRDe, PREn vs. PREe, and CARDn vs. CARDe) and the undivided groups (CKD1-2 and HV), the mean is represented by a more saturated dot, with error bars indicating the standard deviation for each group or subgroup. Note that mean values for the entire ESRD, PRE, and CARD groups are not provided. Additional lines connecting the means are included to enhance plot readability. All Mann–Whitney U tests for the pairs of subgroups with normal and elevated IMT readings were insignificant.

Figure 9 illustrates differences in levels of inflammation markers (hsCRP: Figure 9A, neopterin: Figure 9B, and IL-18: Figure 9C), showing their variability and patterns in different groups. Although no significant difference was detected for inflammation markers in the context of normal and elevated IMTs, some inter-group patterns were visible. First of all, dialyzed patients (ESRD) with an elevated IMT seemed to have higher neopterin levels (Mann–Whitney U = 787, *p*-value = 0.0458); however, it can be argued that this effect may be related to dialysis alone. hsCRP and IL-18 seemed to be affected by the interplay of both CKD and CVD; however, the type of these relationships could not be determined since the IMT was not able to split CKD patients into subgroups related to cardiovascular conditions.

**Figure 10:**
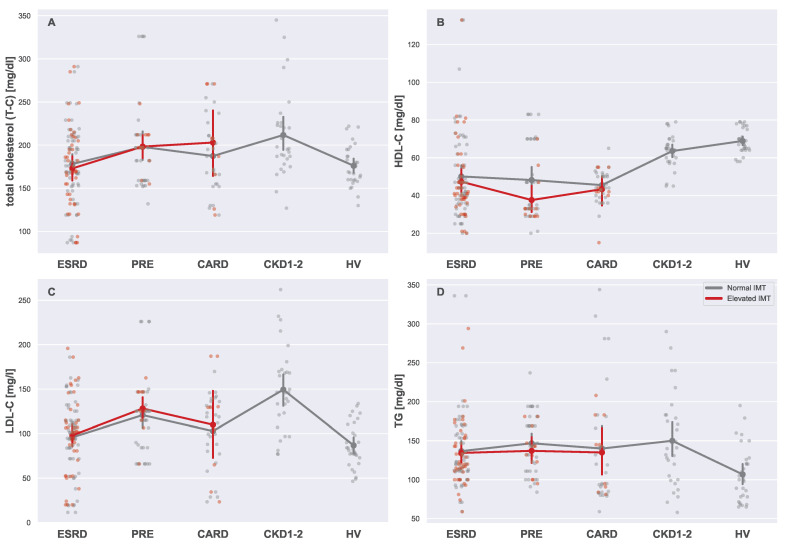
Strip plots presenting differences in lipid profiles ((**A**) total cholesterol, (**B**) HDL-C, (**C**) LDL-C, and (**D**) TG). Each plot displays five main groups: ESRD, PRE, CARD, CKD1-2, and HV. Subgroups with normal and elevated IMTs are distinguished using different colors (gray for normal and red for elevated). For each subgroup pair (ESRDn vs. ESRDe, PREn vs. PREe, and CARDn vs. CARDe) and the undivided groups (CKD1-2 and HV), the mean is represented by a more saturated dot, with error bars indicating the standard deviation for each group or subgroup. Note that mean values for the entire ESRD, PRE, and CARD groups are not provided. Additional lines connecting the means are included to enhance plot readability. All Mann–Whitney U tests for the pairs of subgroups with normal and elevated IMT readings were insignificant.

It is widely assumed that blood lipids are associated with cardiovascular disease. For all lipid markers in this study, significant relations with both CVD and CKD were detected. Unfortunately, the lipid profiles of patients with normal and elevated IMTs did not differ. In fact, such an outcome is not surprising since all patients were properly medicated. Nonetheless, results for lipids presented in Figure 10 show variability between the groups. The highest average levels of total cholesterol (Figure 10A), HDL-C (Figure 10B), LDL-C (Figure 10C), and TG (Figure 10D) were detected in the CKD1-2 group, plausibly due to a lack of deployed treatment or its visible effects. No other patterns were visible for lipid serum markers.

**Figure 11:**
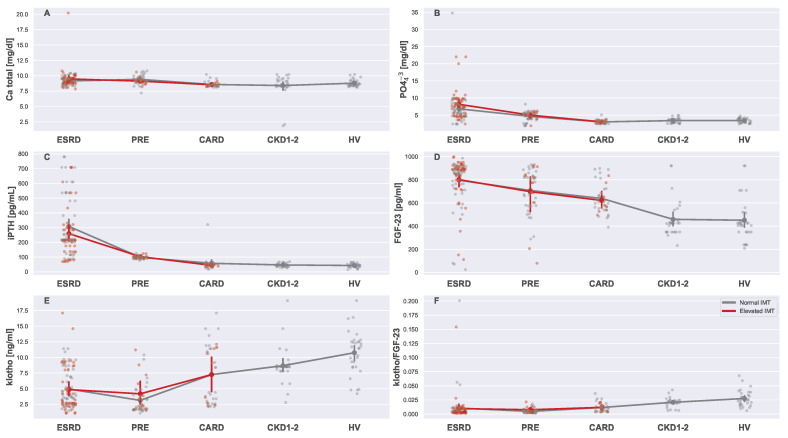
Strip plots presenting differences in calcium (**A**), phosphates (**B**), intact parathyroid hormone levels (**C**), FGF-23 (**D**), klotho (**E**), and klotho/FGF-23 (**F**). Each plot displays five main groups: ESRD, PRE, CARD, CKD1-2, and HV. Subgroups with normal and elevated IMTs are distinguished using different colors (gray for normal and red for elevated). For each subgroup pair (ESRDn vs. ESRDe, PREn vs. PREe, and CARDn vs. CARDe) and the undivided groups (CKD1-2 and HV), the mean is represented by a more saturated dot, with error bars indicating the standard deviation for each group or subgroup. Note that mean values for the entire ESRD, PRE, and CARD groups are not provided. Additional lines connecting the means are included to enhance plot readability. All Mann–Whitney U tests for the pairs of subgroups with normal and elevated IMT readings were insignificant.

The boxplots in Figure 11 are dedicated to calcium and phosphate metabolism parameters. Total calcium, phosphates (${\mathrm{PO}}_{4}^{3-}$), and intact parathyroid hormone (iPTH), as well as FGF-23 and klotho, did not differ significantly between subgroups with normal and elevated IMTs. However, iPTH, phosphate, and calcium correlated only with the renal disease progression, while FGF-23 and klotho were affected by CKD and CVD.

**Figure 12:**
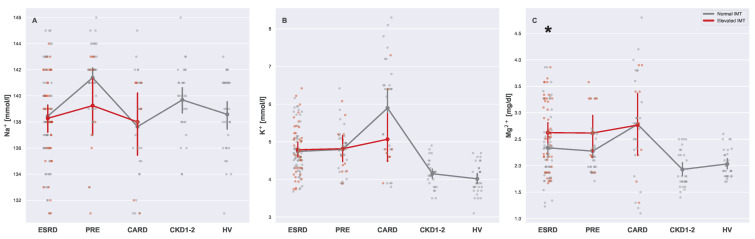
Strip plots presenting differences in blood levels of sodium (**A**), potassium (**B**), and magnesium (**C**). Each plot displays five main groups: ESRD, PRE, CARD, CKD1-2, and HV. Subgroups with normal and elevated IMTs are distinguished using different colors (gray for normal and red for elevated). For each subgroup pair (ESRDn vs. ESRDe, PREn vs. PREe, and CARDn vs. CARDe) and the undivided groups (CKD1-2 and HV), the mean is represented by a more saturated dot, with error bars indicating the standard deviation for each group or subgroup. Note that mean values for the entire ESRD, PRE, and CARD groups are not provided. Additional lines connecting the means are included to enhance plot readability. The pairs of subgroups with normal and elevated IMT readings were annotated with the Mann–Whitney U test significance level (*: 0.05 > *p*-value ≥ 0.01).

No clear pattern for CVD was found for sodium levels in the analyzed groups (Figure 12A). However, more CKD patients exhibited great variability in this context. Potassium levels were high in groups with advanced CKD (ESRD and PRE) as well as in the CARD group (Figure 12B). However, magnesium levels differed in ESRD patients with elevated and normal IMTs. Since the difference was not visible for the cardiovascular patients, it is possible that a poor cardiovascular condition in CKD patients resulted in higher magnesium levels (Figure 12C).

**Figure 13:**
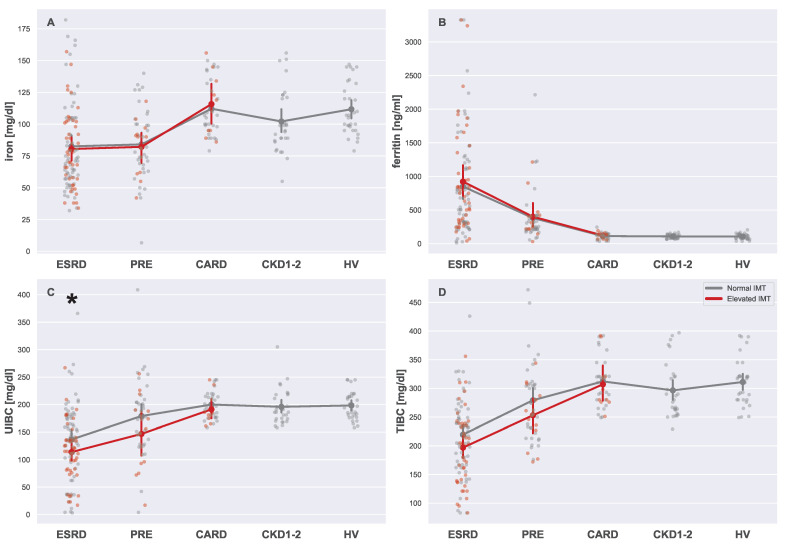
Strip plots presenting differences in iron (**A**), ferritin (**B**), UIBC (**C**), and TIBC (**D**). Each plot displays five main groups: ESRD, PRE, CARD, CKD1-2, and HV. Subgroups with normal and elevated IMTs are distinguished using different colors (gray for normal and red for elevated). For each subgroup pair (ESRDn vs. ESRDe, PREn vs. PREe, and CARDn vs. CARDe) and the undivided groups (CKD1-2 and HV), the mean is represented by a more saturated dot, with error bars indicating the standard deviation for each group or subgroup. Note that mean values for the entire ESRD, PRE, and CARD groups are not provided. Additional lines connecting the means are included to enhance plot readability. The pairs of subgroups with normal and elevated IMT readings were annotated with the Mann–Whitney U test significance level (*: 0.05 > *p*-value ≥ 0.01).

As presented in Figure 13A, lower blood iron levels, as well as increased levels of ferritin, were clearly related only to renal function. In these cases, no differences between subgroups with normal and elevated IMTs were detected (see Table A1). On the other hand, UIBC and TIBC presented much more complex images. Iron binding capacity seemed to be more affected in ESRD and PRE patients, especially for those whose IMT was high. Therefore, it is plausible that in the case of CKD patients, UIBC and TIBC were conditionally affected by CVD advancement.

**Figure 14:**
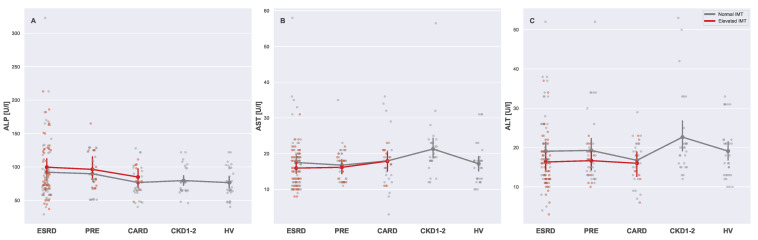
Strip plots presenting differences in liver-related serum markers: alkaline phosphatase (**A**), aspartate transaminase (**B**), and alanine transaminase (**C**). Each plot displays five main groups: ESRD, PRE, CARD, CKD1-2, and HV. Subgroups with normal and elevated IMTs are distinguished using different colors (gray for normal and red for elevated). For each subgroup pair (ESRDn vs. ESRDe, PREn vs. PREe, and CARDn vs. CARDe) and the undivided groups (CKD1-2 and HV), the mean is represented by a more saturated dot, with error bars indicating the standard deviation for each group or subgroup. Note that mean values for the entire ESRD, PRE, and CARD groups are not provided. Additional lines connecting the means are included to enhance plot readability. All Mann–Whitney U tests for the pairs of subgroups with normal and elevated IMT readings were insignificant.

Subgroups with normal and elevated IMTs did not differ in a significant way in terms of liver serum markers (Table 4). Alkaline phosphatase (ALP) seemed to be associated only with CVD (not significantly, see Figure 14A), while in fact, its relation with CKD was significant (Kruskal–Wallis H = 10.7519, *p*-value = 0.0295). Moreover, aspartate transaminase (AST), as depicted on Figure 14B, did not reveal any strong relationships. At the same time, alanine transaminase (ALT) was the only parameter that did not significantly differentiate study groups (Kruskal–Wallis H = 7.663, *p*-value = 0.1047), as shown in Figure 14C. Despite that, in all of these three cases, some patterns emerged. Results of GLMs revealed more insight into ALP and ALT (Tables S3 and S4), suggesting independent and conditional relationships, respectively. It is plausible that a higher number of study subjects would sharpen these already unfolding patterns.

**Figure 15:**
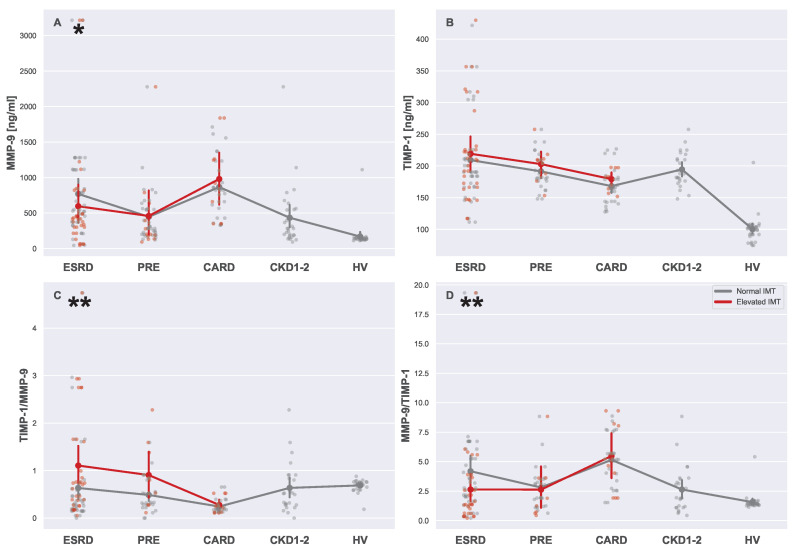
Strip plots presenting differences in metalloproteinases: Matrix metalloproteinase-9 (MMP-9) (**A**), TIMP metallopeptidase inhibitor 1 (TIMP-1) (**B**), TIMP-1/MMP-9 (**C**), and MMP-9/TIMP-1 (**D**). Each plot displays five main groups: ESRD, PRE, CARD, CKD1-2, and HV. Subgroups with normal and elevated IMTs are distinguished using different colors (gray for normal and red for elevated). For each subgroup pair (ESRDn vs. ESRDe, PREn vs. PREe, and CARDn vs. CARDe) and the undivided groups (CKD1-2 and HV), the mean is represented by a more saturated dot, with error bars indicating the standard deviation for each group or subgroup. Note that mean values for the entire ESRD, PRE, and CARD groups are not provided. Additional lines connecting the means are included to enhance plot readability. The pairs of subgroups with normal and elevated IMT readings were annotated with the Mann–Whitney U test significance level (*: 0.05 > *p*-value ≥ 0.01, **: 0.01 > *p*-value ≥ 0.001).

Analyzed metalloproteinase levels (Figure 15) revealed some relationships between MMP-9 and TIMP-1 with CKD and CVD. Both enzymes were definitely related to the CKD stage and kidney function as well as CVD, although these relationships seemed distinct (see Table 4 for reference). Nonetheless, it is worth noticing that while differences related to an elevated IMT in the ESRD group may be a consequence of dialysis treatment (Figure 15A), TIMP-1 was more likely to be independently related to both CKD and CVD (Figure 15B). As a result, the relationships between CKD and CVD with combined parameters, i.e., TIMP-1/MMP-9 and MMP-9/TIMP-1, is challenging to interpret (see Figure 15C,D).

**Figure 16:**
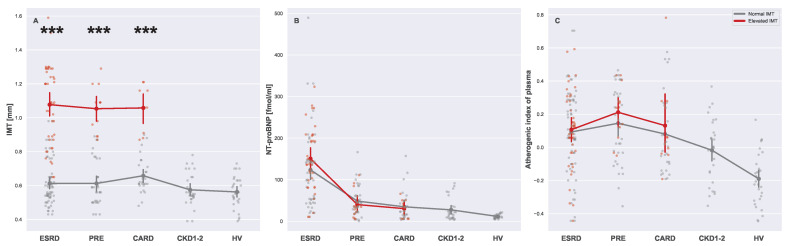
Strip plots presenting differences in levels of cardiovascular markers ((**A**) IMT, (**B**) NT-proBNP, and (**C**) AIP). Each plot displays five main groups: ESRD, PRE, CARD, CKD1-2, and HV. Subgroups with normal and elevated IMTs are distinguished using different colors (gray for normal and red for elevated). For each subgroup pair (ESRDn vs. ESRDe, PREn vs. PREe, and CARDn vs. CARDe) and the undivided groups (CKD1-2 and HV), the mean is represented by a more saturated dot, with error bars indicating the standard deviation for each group or subgroup. Note that mean values for the entire ESRD, PRE, and CARD groups are not provided. Additional lines connecting the means are included to enhance plot readability. The pairs of subgroups with normal and elevated IMT readings were annotated with the Mann–Whitney U test significance level (***: *p*-value < 0.001).

In Figure 16, three cardiovascular markers are presented: IMT (Figure 16A), NT-proBNP (Figure 16B), and AIP Figure 16B. Although the IMT is the factor that separated the groups into subgroups with normal and elevated IMTs, it was presented here to show and a give better understanding of how these groups overlapped since selection criteria were also based on patients’ age. As it can be spotted, groups ESRDn, PREn, and CARDn resembled IMT levels typical for CKD1-2 and the control group. However, this resemblance was not statistically supported (Kruskal–Wallis H = 21.8952, *p*-value = 0.0005), and these groups indeed differed in their IMT level. Moreover, the highest values of the IMT were observed in the ESRDe group. NT-proBNP is an important CV marker. There were no other differences that were statistically significant for NT-proBNP and the AIP in relation to normal and elevated IMT levels. However, NT-proBNP was correlated with CKD progression, while the AIP seemed to present additive and independent effects of both CKD and CVD, especially taking into account medication and the treatment effect on the ESRD group (see Figure 10 on lipids for a reference).

## 4. Discussion

In this study, four groups of patients that differed in CKD stage and in the type of kidney failure treatment, as well as patients with CVD and a control group, were analyzed. The goal of this analysis was to identify markers of increased IMT.

### 4.1. Basic Serum Metabolic Markers

The possible indicators analyzed were basic serum metabolic markers, i.e., glucose, urea, creatinine, eGFR, uric acid, and total protein serum concentration. None of them appeared clearly associated with elevated IMTs in the five studied groups. Glucose levels were significantly higher for patients suffering only from CVD. A clear image of CKD progression was reflected in all other markers (urea, creatinine, eGFR, uric acid, TP).

Although in the case of PRE and CARD groups, urea levels significantly differed in patients with normal (expected) and increased IMTs, the same effect was undetected in ESRD patients. Generally, urea, a principal nitrogenous waste product of metabolism generated from protein breakdown, is well known to be related to CVD [21]. Increased urea levels can lead to molecular changes related to insulin resistance, ROS generation, apoptosis, and disruption of the intestinal protective barrier [22,23]. The results of a prospective epidemiological study [24] indicated that serum urea concentration was associated with CVD and mortality in non-dialyzed patients with CKD, regardless of renal function. An analysis of 2,507 patients with CKD showed that people with higher serum urea concentrations had a higher risk of CV complications and death. Urea in CKD patients directly increased ROS and oxidative stress levels in various cell types [25]. Urea-induced ROS may contribute to the accelerated aging of endothelial progenitor cells, which play a key role in the repair and maintenance of the vascular system. Hence, these vascular repairing processes are affected, predisposing to atherosclerosis. Moreover, there is increasing evidence of indirect toxic effects of urea through post-translational protein modifications. Moreover, BUN, a serum byproduct of protein metabolism, was found to be a valuable predictive CVD biomarker in older people [26].

Our results regarding CIMT and urea in pre-dialyzed and CVD patients without CKD are consistent with the current knowledge; however, deeper analyses are needed in this context to find the mechanisms, especially if kidney function is unaffected or only slightly affected and urea is elevated. Our study was unable to discover this. Still, it seems that even a slightly increased urea serum concentration may indicate the need for further diagnostics for an increased IMT, because it directly and indirectly disturbs several important biochemical functions.

### 4.2. Serum Markers of Oxidative and Nitrosative Stress

In CKD, one of the causes of accelerated atherosclerosis is enhanced oxidative stress and insufficient effectiveness of antioxidant systems. Redox imbalance negatively affects the arterial endothelium and all components of the kidney, contributing to CKD and CVD progression. In addition to oxidative stress, the role of nitrosative stress is emphasized, and both of them contribute to endothelial dysfunction and oxidative damage to lipids, proteins, and DNA in vascular endothelial cells. They are associated with local inflammation and may aggravate atherosclerotic-related disturbances [27]. In our previous study, we confirmed that [28] and discovered that 3-NT, a representative molecule of nitrosative stress, could be a promising marker of 2-year follow-up CV mortality.

Based on the results of this current study, we found that levels of all oxidative and nitrosative stress differed largely in patients with and without CKD. CML, CEL, and MG are reactive compounds formed through glycation and oxidation processes that accumulate in tissues and circulation, contributing to endothelial dysfunction, vascular stiffness, and atherosclerosis in CVD, as well as renal fibrosis and impaired kidney function in CKD [29,30]. None of these compounds was connected to an elevated IMT. This was surprising, since other researchers reported that these markers were related to the IMT [30,31]. According to our results CML and MG were both related to all stages of CDK and CVD, showing a cumulative effect. A similar but different image was reflected by CEL, which behaves like an advanced CKD marker, since its levels were higher only in PRE and ESRD groups.

According to multiple studies, advanced glycation end-products (AGEs) and sRAGE play critical roles in the pathophysiology of cardiovascular disease CVD and chronic kidney disease CKD [32,33,34]. It is believed that dysregulation of these markers is associated with increased inflammation, oxidative stress, and progression of both diseases. The exact image of AGEs and sRAGEs’ relationship with CKD and CVD is up to this day debated, e.g., Caldiroli et al. showed that RAGEs’ isoforms were related independently to malnutrition, while AGEs/sRAGEs balance may be connected to inflammation and oxidative stress [33]. The results obtained in this work for late-stage CKD patients showed that AGEs, but not sRAGEs, were affected by an elevated IMT. Although both AGE and sRAGE levels were higher in CKD and CVD, the PRE and ERSD patients with an elevated IMT had a higher AGEs-to-sRAGEs ratio, when compared to patients with a normal IMT from the same groups. This finding suggest that late-stage CKD patients with an elevated IMT can be related to increased oxidative stress levels and endothelial dysfunction [34,35].

The difference in levels of 3-NT, AOPPs, and MPO were the most significant in the group of dialyzed patients (ESRD), i.e., the levels of these markers were elevated in patients with an elevated IMT. In contrast to 3-NT, the level of MPO was increased both in hemodialyzed patients and peritoneal-dialyzed patients with an elevated IMT. All three markers seemed to be affected by both CKD and CVD, apparently each in a different way. Higher levels of 3-NT seemed to present in late stages of CKD, but only if the IMT was also elevated. The combined effects of CDK and CVD seemed to affect AOPPs, where the strongest effect was observed in ESRD patients, which may suggest some relation to the dialysis treatment itself. These were the only indicators of an increased IMT in all dialyzed patients. Finally, the MPO levels were the highest in the CARD group, suggesting that this is a marker more related to CVD issues. However, since MPO levels were higher in CKD1-2, it can be hypothesized that MPO is independently related to CKD and CVD.

3-NT is a marker related to CKD and CVD simultaneously. It was shown that rats that underwent five-sixth kidney nephrectomies suffered from NO-mediated vasoconstriction. Rats with partly removed kidneys had significantly higher levels of 3-NT, concentrated mainly in the kidneys, but also in the heart, aorta, brain and liver. This was correlated with a decrease in inducible nitric oxide synthase (iNOS) and endothelial NOS [36]. Moreover, higher 3-NT levels are linked to carotid plaque instability [37]. Therefore, it is possible that elevated 3-NT levels relates to both kidney damage and cardiovascular issues. Indeed, our previous study showed that there was a strong correlation between high 3-NT levels and the death of hemodialyzed patients [28].

Similar to 3-NT, relationships were reported for AOPPs. The progression of CKD correlated clearly with the increase in AOPP levels. Moreover, high numbers of AOPPs are related to a faster progression of arteriosclerosis in CKD patients as well as a higher number of calcified atherosclerotic plaques [38,39]. The results obtained in this work align with conclusions made by other researchers, e.g., [38].

Focusing on MPO, an enzyme derived from leukocytes, with hydrogen peroxide and halogen, it is worth emphasizing that many studies have detected a link between its serum concentration and CVD [2,40]. MPO activation enhances protein oxidation (AOPP) and nitration (3-NT). Overall, the presence and activity of MPO in human atherosclerosis are well established, and MPO colocalizes with macrophages in human atherosclerotic lesions [41]. The release of MPO and the formation of the resulting reactive species (MPO-derived reactive species) can be induced by multiple factors. One of these is inflammation, commonly known to cause the recruitment and activation of white blood cells. However, an influx of monocytes also develops into resident macrophages in CKD, some of which express MPO. This expression may be caused by minimally modified LDL particles in the intima. Although LDL cholesterol is generally not elevated in CKD, a higher prevalence of LDL particle modifications is noticed. These modified particles are more easily oxidized and penetrate into the endothelial wall; hence, they are more atherogenic, and thus, subjects who have higher, smaller, and denser lipoproteins are at higher atherogenic risk. These circulating neutrophils are attracted to and associated with sites of damaged endothelium. MPO released by these adherent leukocytes is initially attached to the vascular endothelium and then transcytosed into the subendothelial matrix. Thus, sources of MPO in the vascular wall include both the local release by resident macrophages and the transcytosis of MPO, which is produced intraluminally [42,43]. The study by Mathew et al. demonstrated, for the first time, a causal relationship between bone-marrow-derived MPO and CKD-accelerated atherosclerosis among mice [43]. Studies performed by the same group showed that increased MPO originating from damaged macrophages colocalized with 3-NT and o,o’-tyrosine residues in atherosclerotic plaques in five-sixth-nephrectomized mice, a mouse model of CKD atherosclerosis; unlike non-CKD mouse models of atherosclerosis. This MPO was the source of all three oxidatively modified tyrosines in atherosclerotic lesions [44]. Hence, the researchers suggested that MPO expression and activity may play an essential role in the propagation of atherosclerotic lesions in CKD mice. The key limitation of their work was that the evidence was associative and did not unequivocally demonstrate the causal role of macrophage-derived MPO in CKD-accelerated atherosclerosis. Hence, MPO-increased activity related to the IMT changes among dialyzed patients, observed in our study, was in line with the mentioned results among mouse models.

Protein carbamylation is common in both CKD and CVD. In CVD, elevated levels of carbamylated proteins are linked to increased inflammation, oxidative stress, and adverse cardiovascular events [45,46]. Carbamylated albumin is also associated with the progression of renal impairment and cardiovascular mortality in CKD patients [47]. Our result showed that levels of carbamyl protein groups were high in both CKD and CVD patients, suggesting a cumulative effect in late CKD patients. Interestingly enough, the relationship between protein carbamylation and an elevated IMT was undetected.

### 4.3. Serum Markers of Inflammation

From the studied serum inflammatory markers (hsCRP, IL-18, neopterin) none of them were significant predictors of increased IMT. All markers were related to CKD stages; however, only hsCRP and IL-18 seemed to reveal relationship with CKD. Neopterin levels were the highest in the ESRD group, which may be attributed to the dialysis treatment itself.

To understand this finding, we must first focus on the processes affecting neopterin synthesis and its function. Since interferon-gamma (IFN-γ), released by activated T-helper subtype 1 lymphocyte and natural killer cells stimulates neopterin synthesis by human monocytes and macrophages [48] and also affects the release of ROS from immunocompetent cells, the concentration of neopterin may be an indirect marker of the immunologically induced oxidative stress. A high concentration of neopterin may be a stimulator of increased expression of the nitric oxide synthase (iNOS) gene, leading to increased nitric oxide (NO) production in smooth muscle cells and indicating the role of neopterin as a strong modulator of oxidative stress and the development of atherosclerosis. In ischemic heart disease, neopterin is considered a determinant of damage to the vascular endothelium as a result of increased activity of cells of the monocytic line [49]. Increased levels of circulating neopterin confirm the effect of activated macrophages on vascular regeneration, which in turn may promote nitro-oxidative stress [50] that precedes many vascular pathologies [51]. In CKD, renal dysfunction and immunological disturbances can impact neopterin synthesis [52].

Summarizing the observed relationship between IMT and neopterin in hemodialyzed patients, we revealed that immunological disorders played an extremely important role in the observed multifactorial atherosclerosis, especially among hemodialyzed patients. These are mainly changes in the adaptive immune response associated with uremia. HD and PD treatments themselves and the cause of CKD influence T-cell activation status. Moreover, T-cell-dependent proliferation and differentiation of B lymphocytes are impaired, leading to further disturbances in this group, see [53].

### 4.4. Lipid Profile

All lipid profile markers were related to CKD and CVD. However, there were no significant differences in levels of blood lipids in patients with normal and elevated IMTs. This result did not surprise us, but it made us realize that cholesterol itself was not always the causative factor responsible for endothelial dysfunction and atherosclerosis because we know the innate immune system treats the accumulation of LDL-C in the plasma as an adverse event. Therefore, the endothelial inflammatory response is stimulated to reduce risk by removing excess LDL-C and oxidized LDL-C from the bloodstream to the endothelium, where migrating monocytes take them up for eventual removal [54]. Consequently, the crucial issue here is not cholesterol per se but the inflammatory reaction in response to its accumulation that modifies it with oxidative stress [10].

### 4.5. Serum Calcium, Phosphates, iPTH, Klotho, and FGF-23

Among the tested parameters of calcium metabolism (calcium, phosphates, and iPTH), none of them were associated with an elevated IMT. Although, it was to some extent surprising, all of the mentioned serum markers were associated with CKD. The CKD–mineral bone disorder (CKD-MBD) is a significant complication among dialyzed patients. One of its main features is the abnormal iPTH metabolism, so patients may have either low iPTH levels or secondary hyperparathyroidism. Nevertheless, both endocrine abnormalities are associated with higher CV and all-cause mortality [55,56].

In CKD, reduced renal function leads to decreased klotho expression and elevated FGF-23 levels. This dysregulation contributes to phosphate retention, secondary hyperparathyroidism, and vascular calcification, causing further cardiovascular complications [57]. Low klotho levels are associated with increased oxidative stress, inflammation, and accelerated aging-like phenotypes [58], while elevated FGF-23 independently predicts left ventricular hypertrophy and mortality in CKD patients [59,60]. The imbalance in the klotho/FGF-23 axis was clearly visible in our results, fully agreeing with literature on this topic.

### 4.6. Serum Sodium, Potassium, and Magnesium

Sodium imbalance, particularly hypernatremia and fluid overload, contributes to hypertension and left ventricular hypertrophy in both conditions [61,62], while sodium retention worsens renal damage in CKD, and both hyponatremia and hypernatremia are associated with the death of CKD patients (forming a U-shaped distribution) [62]. This is probably the reason why no clear patterns were detected in this study, as sodium and levels of this ion were greatly diversified in patients within the ESRD, PRE, and CARD groups.

Potassium dysregulation, including hyperkalemia, is a common complication in CKD due to impaired excretion [63] and is linked to life-threatening arrhythmias and sudden cardiac death in CVD [64]. Both advanced CKD and CVD patients have significantly higher potassium level, including notable portions of subjects with hyperkalemia in the CARD, PRE, and ESRD groups. Since the IMT is not related to potassium levels, the relation type between CKD and CVD could not be identified for this ion.

However, the most interesting patterns can be attributed to magnesium. According to the literature, hypomagnesemia is associated with increased risk of arrhythmias, hypertension, and vascular calcification in CVD [65], while low magnesium levels exacerbate oxidative stress and inflammation in CKD [66]. Nonetheless, advanced CKD patients are at high risk of developing hypermagnesemia instead of a magnesium deficiency due to renal malfunction (according to Spiegel, ESRD is the only condition during which hypermagnesemia may be sustained [67]). Indeed, the results in this work show that almost half of the advanced CKD patients suffered from high magnesium levels; interestingly enough, many CVD patients had hypermagnesemia too (>2.6 mg/dL). In addition to that, it seems that magnesium levels were conditionally elevated in CKD patients with a higher IMT, suggesting a positive relationship between magnesium and IMT. Since hypomagnesemia is more often associated with bad consequences to cardiovascular health [65,68], this finding seems to be especially intriguing.

### 4.7. Iron Related Serum Markers

Iron, ferritin, unsaturated iron-binding capacity (UIBC), and total iron-binding capacity (TIBC) are closely linked to CVD and CKD through their roles in iron metabolism and oxidative stress regulation [69]. Iron deficiency and dysregulation are common in CKD due to reduced erythropoietin production and chronic inflammation [70]. The levels of iron in this study revealed iron depletion in the large majority of all CKD patient groups; moreover, a significant fraction of ESRD patients suffered from iron-deficiency anemia. Based on these results, iron levels seemed to be only connected with CKD, since CARD patients did not differ significantly from HV, and iron level did not correspond to an elevated IMT. This finding can be expected, since it is widely believed that inappropriate levels of iron are more likely to cause cardiovascular issues, rather than be the consequence of them [71].

Elevated ferritin levels are often associated with inflammation and oxidative stress [72]. Although this is the more general case, ferritin often acts as an iron buffer, protecting the organism from both iron excess and dearth. Therefore, the main reason why all CKD patient groups had high serum ferritin levels was probably a compensation effect related to iron depletion [73].

UIBC and TIBC reflect iron availability and transport capacity, where low levels can indicate iron overload, further increasing oxidative damage and contributing to vascular injury [74]. While the CARD patients seemed to possess unaffected transferrin levels, all CKD patients showed progressing signs of functional iron deficiency (low TIBC, high ferritin [75]). In addition to that, UIBC levels in late-stage CKD patients were negatively affected by an elevated IMT. This suggests that coexisting CVD worsens functional iron deficiency in CKD patients (this effect was not observed for CVD patients without CKD).

### 4.8. Serum Liver Markers

Liver serum markers, i.e., alanine aminotransferase (ALT), aspartate aminotransferase (AST), and alkaline phosphatase (ALP), are increasingly recognized for their associations with both CKD and CVD. ALP is a marker of vascular calcification and arterial stiffness, contributing to adverse CV outcomes [76]. Although the observed differences in ALP levels between higher and normal IMT subgroups were not significant, they were clearly visible. Since the difference between subgroups with normal and elevated IMTs was more or less constant, and the levels of ALP increased along with the CKD stage, it is plausible that CKD and CVD independently affected ALP serum levels. The relationship between ALP and IMT was clearly visible in this study and may directly explain differences in ALP distributions between study groups. It is well proven that ALT is a good death predictor in various types of patients, including those with CKD [77], signifying systemic inflammation, metabolic syndromes, and liver malfunction. In addition to that, AST is also often linked with CKD [78,79]. Both AST and ALT revealed a similar pattern (unconfirmed, but observable for AST) of conditional dependence on CVD in CKD patients, where ALT and ASP could characterize the IMT state, which was not possible for the typical cardiovascular patients (CARD).

### 4.9. Serum Metalloproteinases

Matrix metalloproteinase-9 (MMP-9) and tissue inhibitor of metalloproteinase-1 (TIMP-1) are key regulators of extracellular matrix (ECM) remodeling [80]. Both of them play a significant role in the progression of CVD. Elevated levels of MMP-9 may be linked to plaque rupture and a fatal acute coronary syndrome (ACS) event, especially if this co-occurs with low TIMP-1 levels, which play a role of natural MMP inhibitor [81]. The presented image overlaps fully with values detected in CARD, where MMP-9 levels were high and TIMP-1 relatively low, which was clearly visible for the MMP-9/TIMP-1 and TIMP-1/MMP-9 juxtapositions. In general, CKD patients exhibited higher levels of TIMP-1 and lower levels of MMP-9 when compared to CARD patients. Nonetheless, levels of both serum markers were much higher in all CKD patient groups than in HV. It is worth noticing that in principle, the creation of subgroups with normal and elevated IMTs should be reflected in MMP-9 and TIMP-1 levels. This was not always the case. Indeed, there was a visible but insignificant pattern for TIMP-1, where patients with elevated IMT possessed higher TIMP-1 levels. However, true differences emerged when TIMP-1 and MMP-9 were analyzed in proportion to each other. TIMP-1/MMP-9 showed clearly that CKD patients differed uniquely from CVD patients, since TIMP-1 levels for CKD patients tried to balance out MMP-9 levels, and it was observed CVD patients possessed the highest concentration of MMP-9 for respective TIMP-1 levels (MMP-9/TIMP-1). Therefore, it is easy to differentiate between CKD and CVD patients based on relative TIMP-1 and MMP-9 values.

### 4.10. IMT, Serum NT-proBNP, AIP

The intima-media thickness (IMT) is a widely used, non-invasive measure of the thickness of the carotid artery walls. The IMT can be easily assessed through ultrasound imaging and serves as a valuable marker for evaluating cardiovascular risk. An elevated IMT reflects structural changes in the arterial wall associated with atherosclerosis and early vascular remodeling, even before clinical symptoms of CVD manifest. Our study discusses a particular use of the IMT as a marker of CV risk in various stages of CKD. Unfortunately, many serum markers did not correlate with the IMT, which left the CKD–CVD relationship detectable but difficult to characterize. Nonetheless, the IMT was successfully used to evaluate the CV risk associated with diseases like cancer [82], diabetes [83], autoimmune [84], stem-cell related [85], or infectious diseases [86].

In our study, NT-proBNP, a recognized marker of heart failure, released from the myocardium in response to increased load on the heart wall due to volume and pressure overload, was the highest in dialyzed patients with an elevated IMT. NT-proBNP is a known reliable predictor of MI in dialysis patients [87]. Moreover, a similar relation between the IMT and NT-proBNP was observed in patients with retinal vein occlusion [88]. More studies are needed to explore whether this relation is causal or only based on similar processes and caused by similar factors.

It has been shown that the Atherogenic Index of Plasma (AIP) can be a good predictor of early-stage CKD [89] as well as the risk associated with CVD [90]. The results obtained in this work support previous findings. The mean values of the AIP in the CKD1-2 and CARD groups were significantly higher than the average value calculated in the HV group. Even higher mean AIP values were observed for the PRE and ESRD groups. Although, the relationship between the AIP and the IMT was visible in the case of the PRE and CARD groups, this was not supported by our statistical analysis. In all cases, it is worth remembering that serum levels of TG and HDL-C might be affected by the treatment and medication administrated to patients.

### 4.11. Limitations and Perspectives of the Study

The small group size was a shortcoming of this study. Moreover, not every stage of CKD was properly represented; CKD 3a stage patients were not taken into account in this study, although CKD stage 3b patients were present, therefore tracking progress of CKD was assured. Moreover, the influence of drugs was not taken into account here, but with this assumption, we performed within-group analyses, not between group ones. In addition, it should be emphasized that we did not examine the quality of the plaque, which would certainly have great cognitive value. We assumed that the IMT was more accessible and possible to determine in clinics. Of course, further studies should be planned to explore this topic better, considering the fact that the IMT test is often performed in hospitals.

A valuable complement to the presented results would be the assessment of the morphology of atherosclerotic plaques, for example, using carotid magnetic resonance imaging. If the IMT value could be associated with biomarkers in blood serum so that a combination of them reflects what is happening in the atherosclerotic plaque, it would allow a better understanding and prediction of plaque dynamics. It would be an interesting alternative to magnetic resonance and other assessments focusing on the quality of the atherosclerotic plaque, which are not always available and performed. For example, machine learning methods could determine such a combination of selected serum markers, the IMT value, and other characteristics of the studied patients.

Results obtained in this way, even before clinical symptoms’ manifestation in patients, could indicate an unstable atherosclerotic plaque or a plaque at high risk of being unstable. This approach would point out the necessity for more comprehensive assessments, especially among selected patients.

## 5. Conclusions

This study analyzed an association between the biomarkers related to metabolism, oxidative stress, lipids, inflammation, metalloproteinases, calcium–phosphate balance, ions, iron, and CV risk. The study compared patients at different stages of CKD: CKD1-2, PRE (CKD stages 3b-4), and ESRD (CKD stage 5) with subjects suffering from cardiovascular disease but without chronic kidney disease and healthy people as a control group.

The main goal of this study was to untangle possible relationships between CVD and CKD, assuming that the IMT was a good CV risk marker. The results showed that glucose was the only marker that could be attributed to CVD alone. The following markers were found to be associated only with CKD: urea, creatinine, eGFR, total protein, CEL, neopterin, total calcium, phosphates, iPTH, sodium, iron, ferritin, and AST. The interplay of CKD and CVD was present in all remaining markers. Based on a comparison of patients with normal and elevated IMTs, in some cases, it was possible to determine a plausible type of CKD–CVD relationship, i.e., independent (effects of CKD and CVD are additive): MPO, ALP, MMP-9, and MMP-9/TIMP-1; combined (CKD and CVD, acting together, have an enhanced effect on a marker due to their interaction): AOPPs and TIMP-1; conditional (the CVD effect is specific only to CKD patients): AGEs, 3-NT, magnesium, UIBC, TIBC, ALT, and TIMP-1/MMP-9. Since it was not possible in all cases to use the IMT as a differentiating factor related to CV risk, the following markers were proven to be related to both CKD and CVD, but the nature of such interaction could not be determined: CML, sRAGEs, carbamyl protein groups, protein carbamyl, hsCRP, total cholesterol, HDL-C, LDL-C, TG, IL-18, klotho, FGF-23, klotho/FGF-23, potassium, NT-proBNP, and AIP.

In general, results presented in this work showed that relations between IMT and serum biomarkers were not trivial. The carotid IMT is not always a good predictor of CV risks in CKD patients, and perhaps a more detailed analysis of arterial walls needs to be deployed in order to properly assess CV state. A good candidate for enriching the standard IMT assessment would be an additional arterial tonometry analysis [9] or new imaging methods able to evaluate the progression of arterial wall thickening with respect to its composition [91].

## Figures and Tables

**Table 1:** Separation criteria for creating subgroups of patients with normal and elevated IMT. The table presents the age-dependent IMT thresholds that were used for patient grouping. The values are taken from Engelen et al. [12].

Age Range [years]	Maximal Normal IMT [mm]
<30	0.645
>30 and <40	0.714
>40 and <50	0.784
>50 and <60	0.854
>60 and <70	0.89
>70	0.989

**Table 2:** Basic information about the groups (MEAN ± SD or %) on the duration of dialysis, percent of deceased patients during the two-year follow-up, and months lived before death (for patients that died during the two-year follow-up).

	Duration of Dialysis [months]	(% Dead)	Months Lived
ESRD (n = 106)	19.63 ± 22.64	28.17%	13.46 ± 5.72
PRE (n = 48)	-	10.42%	15.4 ± 5.5
CARD (n = 37)	-	18.92%	12.86 ± 5.21
CKD1-2 (n = 29)	-	10.34%	16.33 ± 5.03
HV (n = 31)	-	0%	-

**Table 3:** The sizes of subgroups obtained by separating patients with normal and high IMT levels.

Group	Number of Patients with Normal (Expected) IMT	Number of Patients with Elevated IMT	Total
ESRD	63	43	106
PRE	36	12	48
CKD1-2	29	0	29
CARD	28	9	37
Control (HV)	31	0	31

**Table 4:** Kruskal–Wallis test results that compare all five groups (not divided based on the IMT), i.e., ESRD, PRE, CARD, CKD1-2, and HV. Identified relationships with CKD, CVD, and IMT were listed in separate columns (notice that the relation with the IMT implies the relation with CVD). Relationship types are described as singular (CKD or CVD but not both), independent (effect of CKD and CVD is additive), combined (effect of CKD and CVD is mutual, one enhances the other), or conditional (effect of CVD is present only in CKD patients). In cases where the IMT was unrelated to a marker, the relationship type between CKD and CVD could not be established (-). Relationship support was described as weak, moderate, or strong based on the results of GLMs (Tables S1–S5, parameters: $R_{adj}^{2}$, AIC, Deviance, and log-likelihood) as well as non-parametric test, i.e., Kruskal–Wallis test in this table and Mann–Whitney U test (Table A1).

Variable	Kruskal–Wallis Test		Identified Relationships				
	H	*p*-Value	CKD	CVD	IMT	Type	Support
Glucose	25.0792	10^−4^	No	Yes	No	Singular	Weak
Urea	154.2593	10^−4^	Yes	No	No	Singular	Strong
Creatinine	218.0032	10^−4^	Yes	No	No	Singular	Strong
eGFR	215.5483	10^−4^	Yes	No	No	Singular	Strong
Uric acid	27.4569	10^−4^	Yes	No	No	Singular	Weak
Total protein	13.2389	0.0102	Yes	No	No	Singular	Weak
CML	80.0525	10^−4^	Yes	Yes	No	-	Moderate
CEL	125.789	10^−4^	Yes	No	No	Singular	Moderate
MG	129.4426	10^−4^	Yes	Yes	No	-	Moderate
AGEs	32.4284	10^−4^	Yes	Yes	Yes	Conditional	Weak
sRAGEs	29.4331	10^−4^	Yes	Yes	No	-	Weak
3-NT	92.0481	10^−4^	Yes	Yes	Yes	Conditional	Moderate
AOPPs	116.9362	10^−4^	Yes	Yes	Yes	Combined	Moderate
Carbamyl protein groups	135.5547	10^−4^	Yes	Yes	No	-	Moderate
Protein carbamyl	20.8522	0.0003	Yes	Yes	No	-	Weak
MPO	31.2417	10^−4^	Yes	Yes	Yes	Independent	Weak
hsCRP	86.6482	10^−4^	Yes	Yes	No	-	Weak
Total cholesterol	15.3171	0.004	Yes	Yes	No	-	Weak
HDL-C	60.1765	10^−4^	Yes	Yes	No	-	Moderate
LDL-C	39.9319	10^−4^	Yes	Yes	No	-	Moderate
TG	19.655	10^−4^	Yes	Yes	No	-	Weak
Neopterin	173.2084	10^−4^	Yes	No	No	Singular	Moderate
IL-18	84.9191	10^−4^	Yes	Yes	No	-	Weak
Ca total	47.1012	10^−4^	Yes	No	No	Singular	Weak
${\mathrm{PO}}_{4}^{3-}$	123.5818	10^−4^	Yes	No	No	Singular	Strong
iPTH	190.9818	10^−4^	Yes	No	No	Singular	Strong
Klotho	83.9677	10^−4^	Yes	Yes	No	-	Moderate
FGF-23	95.4575	10^−4^	Yes	Yes	No	-	Moderate
Klotho/FGF-23	111.592	10^−4^	Yes	Yes	No	-	Moderate
Na^+^	25.5602	10^−4^	Yes	No	No	Singular	Weak
K^+^	76.1068	10^−4^	Yes	Yes	No	-	Moderate
Mg^2+^	37.7502	10^−4^	Yes	Yes	Yes	Conditional	Moderate
Iron	52.4437	10^−4^	Yes	No	No	Singular	Moderate
Ferritin	144.1993	10^−4^	Yes	No	No	Singular	Moderate
UIBC	73.0284	10^−4^	Yes	Yes	Yes	Conditional	Moderate
TIBC	101.2514	10^−4^	Yes	Yes	Yes	Conditional	Weak
ALP	10.7519	0.0295	Yes	Yes	Yes	Independent	Weak
AST	15.2443	0.0042	Yes	No	No	Singular	Weak
ALT	7.663	0.1047	Yes	Yes	Yes	Conditional	Weak
MMP-9	74.5858	10^−4^	Yes	Yes	Yes	Independent	Moderate
TIMP-1	83.2287	10^−4^	Yes	Yes	Yes	Combined	Moderate
TIMP-1/MMP9	38.9971	10^−4^	Yes	Yes	Yes	Conditional	Weak
MMP-9/TIMP-1	41.869	10^−4^	Yes	Yes	Yes	Independent	Weak
IMT	32.7136	10^−4^	Yes	Yes	Yes	Independent	Strong
NT-proBNP	112.6968	10^−4^	Yes	Yes	No	-	Moderate
AIP	48.9072	10^−4^	Yes	Yes	No	-	Moderate

## Data Availability

All necessary data are included in the paper.

## Supplementary Materials

The following supporting information can be downloaded at: https://www.mdpi.com/article/10.3390/biomedicines13020335/s1, Table S1: Only CKD; Table S2: Only CVD; Table S3: Independent effects; Table S4: Combined effects; Table S5: Random effects.

## Appendix A. Supporting Results

**Table A1:** Results of Mann–Whitney U tests performed to compare variables between patients separated into subgroups based on their IMT levels. Since the size of certain groups can be considered small, the critical U statistic values are given for three comparisons: PREn vs. PREe: 100, CARDn vs. CARDe: 48, and the comparison between ESRDn and ESRDe, which is based solely on *p*-value.

Variable	ESRDn vs. ESRDe		PREn vs. PREe		CARDn vs. CARDe	
	U	*p*-Value	U	*p*-Value	U	*p*-Value
Glucose	1622	0.085	201.5	0.738	136	0.733
Urea	1077.5	0.109	288.5	0.024	189	0.027
Creatinine	1455	0.519	288	0.089	122.5	0.915
eGFR	1390	0.821	150	0.118	129	0.929
Uric acid	1474.5	0.441	266.5	0.234	165.5	0.167
hsCRP	1393	0.806	232.5	0.703	92.5	0.242
TC	1247.5	0.492	261.5	0.277	145.5	0.501
HDL-C	1265	0.566	138.5	0.063	129	0.929
LDL-C	1366	0.943	267.5	0.218	132.5	0.832
TG	1347.5	0.966	186.5	0.488	142.5	0.571
total protein	1183	0.27	192	0.572	131	0.872
CML	1267	0.771	242.5	0.536	169	0.132
CEL	1261	0.741	230	0.748	82.5	0.128
MG	1048	0.082	203	0.766	169.5	0.128
AGEs	1551.5	0.114	311	0.024	133	0.818
sRAGEs	1400.5	0.559	264	0.258	63	0.027
3-NT	1721	0.018	256	0.347	128	0.958
AOPPs	1671	0.042	240	0.576	129	0.929
Carbamyl protein groups	1286	0.662	205.5	0.812	153	0.347
Protein carbamyl	1276	0.616	210	0.896	73	0.063
Neopterin	1568	0.17	272.5	0.182	128	0.958
IL-18	1429	0.354	249	0.439	133.5	0.804
NT-proBNP	1078.5	0.069	179	0.385	116.5	0.750
MPO	1859	0.0004	284.5	0.105	154	0.330
Ca total	1460.5	0.497	147	0.1027	137	0.707
${\mathrm{PO}}_{4}^{3-}$	1600.5	0.082	260	0.2986	138.5	0.6685
iPTH	1068.5	0.122	236	0.5337	89.5	0.2482
Klotho	1224.0	0.4027	253.0	0.3836	124.5	0.9717
FGF-23	1491.5	0.3798	239.0	0.5921	116.0	0.7365
Klotho/FGF-23	1205.5	0.3393	258.0	0.3231	138.0	0.6838
Na^+^	1344.0	0.9485	158.5	0.1691	134.0	0.7889
K^+^	1417.5	0.6875	216.0	1.0	75.5	0.0754
Mg^2+^	1656.5	0.0351	277.0	0.1471	120.0	0.9708
Iron	1343.0	0.9436	204.5	0.7933	128.5	0.9435
Ferritin	1467.5	0.4691	244.0	0.5125	145.0	0.5125
UIBC	1030.5	0.0374	169.0	0.2681	100.0	0.3663
TIBC	1073.5	0.071	177.5	0.3655	110.5	0.5949
ALP	1587.5	0.1345	220.5	0.9239	161.5	0.2153
AST	1268.0	0.5789	208.5	0.8658	142.0	0.5821
ALT	1116.5	0.1256	200.5	0.72	118.0	0.79
MMP-9	452.5	0.027	166.0	0.2383	139.5	0.6451
TIMP-1	692.5	0.5201	128.5	0.3292	167.5	0.1463
TIMP-1/MMP9	893.5	0.0069	140.0	0.2949	119.5	0.8316
MMP-9/TIMP-1	408.5	0.0099	84.0	0.428	132.5	0.8316
IMT	2591.5	<10^−4^	429	<10^−4^	248.5	<10^−4^
AIP	1403.5	0.755	245	0.497	146.5	0.479
